# Advancing the Science of Electronic Health Record Transitions

**DOI:** 10.1007/s11606-023-08333-x

**Published:** 2023-10-05

**Authors:** Michael Weiner, Seppo T. Rinne, David A. Haggstrom, Elizabeth M. Yano

**Affiliations:** 1 Center for Health Information and Communication, U.S. Department of Veterans Affairs, Veterans Health Administration, Health Services Research and Development Service CIN 13-416, Richard L. Roudebush VA Medical Center, 1481 West 10Th Street (11H), Indianapolis, IN 46202 USA; 2 Department of Medicine, Indiana University School of Medicine, Indianapolis, USA; 3 Center for Health Services Research, Regenstrief Institute, Inc., Indianapolis, IN USA; 4 Center for Healthcare Organization and Implementation Research, VA Bedford Healthcare System, Bedford, MA USA; 5 Department of Medicine, Boston University Chobanian & Avedisian School of Medicine, Boston, MA USA; 6 VA HSR&D Center for the Study of Healthcare Innovation, Implementation and Policy, VA Greater Los Angeles Healthcare System, Los Angeles, CA USA; 7 Department of Medicine, David Geffen School of Medicine, University of California, Los Angeles, USA; 8 Department of Health Policy & Management, Fielding School of Public Health, University of California, Los Angeles, USA

Many health institutions that have adopted electronic health record systems (EHRs) eventually decide to switch to a different one. The reasons for these changes vary and include cost, system functionality, corporate consolidation, efficiency, safety, federal incentive programs,^1^ and legislation such as the Health Information Technology for Economic and Clinical Health Act.^2^ Regardless of the reason, understanding the tradeoffs and impacts of such a transition is not straightforward, as the technical switch from one EHR system to another is only the tip of the iceberg. An EHR change is a *sociotechnical* phenomenon^3^ that can affect not only clinical outcomes and patient safety, but training and trainees; change management, communication, and institutional culture; preserving quality of care; the feasibility and conduct of research; inter-professional and cross-site coordination; migration, syndication, and validity of data; system performance, maintenance, and optimization; user experience and workflow; and governance of problems and the system itself. Innovation and research are critical to ensure a well-functioning system that can adapt to evolving needs. When the US Department of Veterans Affairs (VA) began its enterprise-wide transition from a longstanding homegrown EHR to a commercial EHR, it had to consider all of these factors. Despite extensive planning and investment into the change, unexpected problems emerged,^4–6^ leading to a safety-based “stop the line” approach in pausing the implementation.

Within an organization, no individual department or group has sufficient advance knowledge to address all areas mentioned above. Through rigorous design and methods, evidence about the factors at hand must be gathered, analyzed, interpreted, and applied, just as they would for other types of major organizational change. Nonetheless, the body of published evidence about EHR transitions is small. In this special issue sponsored by the VA Health Services Research and Development Service, we present 12 research articles and five perspective articles that bring substantive new science, commentary, and knowledge about EHR transitions and their impacts. The settings examined include both VA and non-VA institutions across diverse organizational and geographic settings. Methods are necessarily manifold and include retrospective, prospective, qualitative, quantitative (to a lesser degree), and mixed-methods approaches. This journal issue illuminates both salient findings and knowledge gaps to be explored in future research. EHR transitions may be most successful with careful attention to many of the factors and lessons presented here.

## The Past and Future of Research in EHR Transitions

The journal issue begins with a broad survey of literature about EHR transitions. Building upon previous reviews,^7,8^ Miake-Lye et al. performed a systematic review of 40 publications. The research that they described illustrates the far-reaching impact of EHR transitions throughout an entire organization, and the multi-faceted factors affecting the structures, processes, and outcomes of transition. EHR research itself has benefited from many methodological approaches from areas such as human factors, biomedical informatics, qualitative research, and implementation science. The current review demonstrates the many tradeoffs experienced in practice, as well as heterogeneity in the outcomes and timelines associated with EHR transition. The difficulty of EHR transition for clinicians seems universal, prompting greater efforts to prevent adverse effects especially in the near term. Through stakeholder engagement, Cogan et al. build on these past findings in describing a new and thoughtful research agenda for EHR transitions, taking into account health system priorities and the need to be proactive in optimizing the success of implementation.

## Usability

The usability of EHR systems holds significant importance to research and practice. Essential pursuits of usability include generating evidence-based knowledge about it, determining appropriate usability outcome measures, advocating for improvements, and exploring avenues for enhanced design and functionality beyond vendors’ constraints. This issue addresses several dimensions of usability. Marcilly et al. provide a report of end users conducting a usability “walkthrough” assessment of candidate EHR systems. The premise is that such findings should inform EHR procurement instead of just constituting an exercise to be conducted following implementation. The participants identified hundreds of usability problems. Reale et al. examined bar code medication administration, which poses special challenges for nurses involved in EHR transitions, as EHRs must properly integrate with equipment such as barcode scanners. They reported risks related to nurses’ medication tasks, such as when the user interface required excessive vertical scrolling, obscured windows, or hindered identification of medications that were due. Ahlness, Orlander, et al. explored employees’ roles and responsibilities, including EHR-designated roles, at the VA’s first site implementing a commercial EHR system. They report on the impact of discrepancies between their clinical responsibilities and the EHR features and functionality that they were authorized to access. With the close relationship between professional roles and workflows, this article has implications for those involved in management, supervision, performance, staffing, and role assignments. Molloy-Paolillo et al. assessed usability and EHR usage time, citing concerns from users but also improvements in EHR documentation and order time.

Finally, specialty referral and consultation are fundamental needs for any health institution and EHR. Referral templates are the rule and can augment, or inadvertently supplant, the role of interpersonal communication in the referral process. Cordasco et al. interviewed primary care providers, primary care nurses, and specialty providers involved in referrals following VA’s EHR transition. In addition to proper transmission of information from referrals and consultations, usability issues such as efficiency, simplicity, and consistency were identified as important factors contributing to the magnitude of perceived success with care pathways.

These articles demonstrate the delicate interplay between people and their work environments and systems—the essence of human factors. Institutions considering an EHR transition should consider these highly informative findings, activities, and approaches in their decision-making and planning.

## Governance and Organizational Change

Although computer systems should serve their users, it often feels as though the users must serve the systems. Users’ perspectives are critical in identifying specific problem points that need attention, as well as forecasting success. This issue reports some of the most extensive recent investigations into governance and organizational change surrounding EHR transition. Using qualitative methods, Rinne et al. report users’ perspectives about supportive and successful organizational practices. Their findings will help institutions to maximize their successes in connecting frontline personnel to EHR change agents, administrative and clinical leaders, and others.

One goal of some EHR transitions is the standardization of the system, with accompanying standardization of clinical practice. Brunner, Cannedy, et al. report on the scope, content, and anticipated implications of standardization as experienced in the VA. The findings will help organizations understand the governance process and its effects, as well as how stakeholders have viewed it. In a separate article, Brunner, Anderson, et al. identified perceived impacts of single-vendor integrated EHR systems on the workforce. The apparent role of these systems in diminishing perceptions of professional autonomy is striking and sparks the question of how we will “reverse the tide,” so that EHR systems appropriately foster autonomy, decrease burnout, leverage creative problem solving, speed documentation, and enable health professionals to use their most needed skills more effectively and efficiently—instead of doing the opposite.

## Agile Support for Shifting Conditions and Needs

Trainees are sometimes overlooked in the flurry of activity that surrounds planning for an EHR transition. They are often transient to a setting, by design. Nonetheless, clinical trainees of many disciplines have essential EHR needs and represent the frontline of medical care in many settings. What is the impact of EHR transition on health professions trainees? Ahlness, Molloy-Paolillo, et al*.* examined this question in a formative mixed-method evaluation of trainees’ experiences before, during, and after VA’s EHR transition. They touch on key issues such as on-boarding, clinical care, and VA career retention. Rucci et al. report on the co-occurrence of EHR transition and the COVID-19 pandemic, and how to optimize management of concurrent stressors that may threaten clinical care during such a difficult period for the institution. Their findings of important relationships between the pandemic and the success of EHR transition provide lessons in how to plan for adequate training and support in the face of shifting external factors. The review by Miake-Lye et al. (summarized earlier) touches more broadly on longitudinal changes in outcomes, some positive and some negative, in measures such as safety, noted by some to appear to worsen for a few months, and then improve.^9,10^ Although standardization of the EHR’s features and functions may be a goal, if shifting conditions and needs in the environment can be anticipated, recognized, and measured, then the approach to managing the change can adapt by intent, fostering more successful transitions.

Finally, a set of perspectives from noted experts discuss patient safety, additional factors contributing to successful transition, secondary uses of observational data, roles of research, and the critical need for interventional informatics. This issue of *Journal of General Internal Medicine* offers unique and important insights that can guide readers in their current or upcoming EHR transitions.

## Author Contributors

This journal issue would not have been possible without the many reviewers of the articles herein. In addition, we thank the members of our Guest Editorial Board for their time, skill, and dedication in serving as key experts and guides in our process: Julia Adler-Milstein, Jessica Davila, Christian Helfrich, Sarah Krein, Blake Lesselroth, Michael Matheny, Amanda Midboe, and Hardeep Singh. We thank Isomi Miake-Lye and the editorial staff of the *Journal of General Internal Medicine* for organizational support.
